# Control of Alternaria Leaf Spot of the Common Bean (*Phaseolus vulgaris* L.) Using Soil-Derived Biological Agents

**DOI:** 10.1155/2024/3896663

**Published:** 2024-02-06

**Authors:** Stella Karonji, Nixon Odiwuor Odhiambo, Joshua Kiilu Muli, Julius Mugweru, Romano Mwirichia

**Affiliations:** Department of Biological Sciences, University of Embu, P.O. Box 6-60100, Embu, Kenya

## Abstract

*Phaseolus vulgaris* L. is considered one of the most essential legume crops in Kenya. *Alternaria alternata* is an economically significant plant pathogen that causes Alternaria leaf spot which accounts for over 70% yield losses of beans in Kenya. Chemical fungicides based on copper and sulfur are used to control Alternaria leaf spot in bean plants, but their prolonged use has adversely affected the environment and the health of workers. Herein, we tested the biocontrol potential of bacterial agents from soil planted with Rosecoco bean plants infected with *A. alternata*. Using bacterial suspensions at different time intervals, we evaluated the putative bacterial biocontrol activity against *A. alternata* under greenhouse conditions. *B. subtilis* and *B. velezensis* bacterial biocontrol agents significantly suppressed disease severity by 20% and 21.2% on the 45^th^ day, respectively. Our study demonstrates that *B. subtilis* and *B. velezensis* are promising biocontrol agents that could be integrated in the management of Alternaria leaf spot.

## 1. Introduction

The common bean *Phaseolus vulgaris* is a crop that is planted worldwide. In Kenya, approximately 75% of this crop is grown in the Eastern, Nyanza, and Rift Valley regions which are characterized by well-drained soils, low-altitude areas, with 800–2000 mm rainfall, and an optimum temperature of 20–25°C [1]. GLP X92 (Mwitemania), GLP X1127 (Mwezi Moja), and GLP 2 (Rosecoco) are the varieties that are predominantly grown in these regions [2]. Low soil fertility arising from poor farming systems and biotic factors such as bacterial and fungal diseases are a major challenge to small-holder farmers [3]. Among the fungal diseases that lower the common bean yield in Kenya is Alternaria leaf spot disease caused by the fungus *Alternaria alternata* [4].

*Alternaria alternata* attacks bean leaves causing characteristic small irregular brown lesions which can turn black with centered rings [5]. Spotting occurs on lower and mature leaves, and the lesions may dry and fall off causing a shot-hole appearance on the leaf when the infection is severe or combined to form huge necrotic spots [6]. Heavily spotted leaves may become yellow, wither, and fall due to water deficiency [5]. Management of Alternaria leaf spot in Kenya is carried out using sulfur- and copper-based fungicides like Bonide™, Cuptocaffaro WP, and Cuprocaffaro Micro 37.5 WG, respectively [7]. Sulfur fungicides prevent the spread of fungal spores, and copper ions kill the fungus by denaturing the enzymes and proteins in pathogen cells. Although the sulfur- and copper-based fungicides have been effective in minimizing the spread of Alternaria leaf spot, they irritate the applicants' eyes and skin, can cause bodily harm if ingested, and also pollute the environment [8].

Biological control has been postulated as an alternative method for controlling Alternaria leaf spot because it is eco-friendly and less toxic as compared to synthetic chemicals. Management of disease-causing macroorganisms using rhizobacterial strains has previously been reported [9, 10]. Diverse species groups of microorganisms colonize plant tissues and provide long-term protection throughout the growing period [11, 12]. Disease suppression occurs when the colonizing microorganisms compete for space or nutrients with the pathogen or produce lytic enzymes, siderophores, and antibiotics [13, 14]. Tozlu et al. [15] reported the potential application of *Trichoderma harzianum* and *Bacillus* species isolated from the rhizosphere of tomato plants in controlling *A. alternata.* We hypothesized that some of the microbes within the bean rhizosphere microbiome could have the potential to suppress soil fungal pathogens. In Kenya, these soils are yet to be explored for their potential biocontrol application. Therefore, the aim of this study was to isolate and screen for bacterial antagonists for *A*. *alternata* that infects common beans.

## 2. Materials and Methods

### 2.1. Isolation and Characterization of Fungal Pathogen and Bacterial Biocontrol Isolates

Purposive sampling was conducted at one of the universities of Embu farm plots (0.30′47.0″S 37°27′09.46″E) using GLP 2 bean plants displaying leaf spot symptoms. Leaves were collected and surface-sterilized using the following protocol: 3% bleach plus Tween 20 for 30 seconds, 70% ethanol for 10 seconds, and a thorough rinse using sterile water for 30 seconds. The leaves were cut into thin sections using sterile scalpel blades, macerated in sterile phosphate-buffered saline (PBS), and vortexed to homogenize the solution. The solution was serially diluted, and 100 *μ*L aliquots from dilutions of 10^−1^ and 10^−2^ were plated onto potato dextrose agar (PDA plates) amended with ampicillin (50 *μ*g/mL) to inhibit the growth of bacteria. The plates were incubated at 30°C for 7 days to allow fungal growth. Mycelia were picked based on morphological features and transferred to new PDA plates to obtain pure cultures. We sampled soils from the same bean growing plots because we hypothesized that these soils contain microbes that could suppress fungal pathogens. One gram of soil was mixed with 1 mL of PBS and serially diluted, and 100 *μ*L from dilutions 10^−2^, 10^−4^, and 10^−6^ was spread plated onto Trypticase soy agar plates supplemented with cycloheximide (50 mg/L) to inhibit the growth of fungi and incubated at 30°C for 24 hours. Morphological characteristics such as color, elevation, and form were examined using standard microbiological techniques and recorded as described [16]. Distinct colonies were selected and transferred to new plates to obtain pure cultures.

### 2.2. Antagonism Assays of Bacterial Biocontrols

Single colonies were selected, purified, and screened for ability to inhibit *A. alternata* as follows: Ten microliters of fungal culture broth containing 1 × 10 ^5^ spores was spread plated onto nutrient agar plates, and 10 *μ*L of the bacterial biocontrol was spotted using a sterile pipette. The plates were incubated at 30°C for 24 hours. Zones of inhibition around the bacterial colonies indicated antagonistic activity against *A. alternata*. The inhibition zones measuring 1.5–3.5 mm were considered significant and indicated possible biocontrol agents for *A. alternata*.

### 2.3. Biochemical and Physiological Characterization of Bacterial Isolates

The bacterial isolates were then tested for their ability to utilize starch, carboxymethyl cellulose, cellulose, skimmed milk, tributyrin, citrate, and phosphate polymers by spotting them on nutrient agar media supplemented with respective substrates. The plates were incubated at 30°C for 24 h. Carboxymethyl cellulose and starch plates were flooded with Gram's iodine solution. Formation of clear zones around bacterial growth after staining showed positive enzyme production, while the presence of blue-black color on starch plates indicated the absence of enzyme production. Lipolytic activity was indicated by clear zones around the tributyrate agar, while the absence of clear zones indicated negative results. Citrate utilization was tested through introducing isolates into Simmons citrate agar slants and incubating them at 30°C. Media change from green to blue showed positive results, while media retaining green color indicated negative results. For phosphate utilization, bacterial isolates were inoculated onto media containing phosphate supplemented with bromothymol blue and incubated at 30°C for 48 hours. Formation of clear zones around the bacterial isolates and media color change from green to orange indicated positive results. To understand the optimum growth conditions, the bacterial biocontrol agents were tested for optimum growth at salt concentrations of 5–15%, pH ranges of 5–12, and temperature ranges of 25−45°C. Growth was assessed by measuring OD_600._

### 2.4. Molecular Characterization of Fungal Pathogen and Bacterial Biocontrol Isolates

Genomic DNA was extracted using the phenol-chloroform protocol as described by [17] and used as a template for polymerase chain reaction. PCR amplification of the ITS region was performed using the universal fungal primer pair of ITS1 (5′-TCCGTAGGTGAACCTGCGG-3′) and ITS4 (TCCTCCGCTTATTGATATGC-3′) [18]. Amplicons were generated on Sure Cycler 8800 (Agilent Technologies) in a total reaction volume of 30 *μ*L (22 *μ*L PCR water, 6 *μ*L of polymerase buffer, 0.6 *μ*L of 20 *μ*M ITS 1 primer and 0.6 *μ*L of 20 *μ*M ITS 4 primer, 0.6 *μ*L Taq polymerase (0.5 *μ*M), and 0.2 *μ*L of 50 ng genomic DNA). The PCR conditions were as follows: 45 sec of denaturing at 95°C, 30 sec of annealing at 53°C, and 45 sec of elongation at 72°C followed by a last step of 4 min at 72°C. Amplified products were separated on 1% agarose gel in EDTA buffer and visualized under UV light after staining with a fluorescent dye. The amplified fragments were cleaned by mixing 12.5 *μ*L of the PCR product with 2.5 *μ*L of Exo SAP-IT (Thermo Fisher Scientific) and incubated at 37°C for 30 min followed by heating the mixtures at 95°C for 5 min to stop the reaction [19]. Genomic DNA from the bacterial isolates was extracted and purified by the method described by Chaudhary et al. [20]. The 16S rRNA gene was amplified by primers (27F and 907R) using Sure Cycler 8800 (Agilent Technologies). 30 *μ*L master mix comprising 22 *μ*L PCR water, 6 *μ*L of 5 ×Taq buffer, 0.6 *μ*L of Taq polymerase (0.5 *μ*M), 0.6 *μ*L of 10 *μ*M reverse primer, 0.6 *μ*L of 10 *μ*M forward primer, and 0.2 *μ*L of 50 ng DNA were subjected to 35 cycles of initial denaturation at 95°C for 5 minutes, strand thickening at 45°C for 30 seconds, elongation at 72°C for 45 seconds, and a containment step at 10°C [20]. The PCR amplicons were separated using 1% agarose gel and visualized under UV after staining with a fluorescent dye. The amplicons were sequenced at Inqaba Biotech, South Africa. Sequences obtained were edited using Chromas Lite software. Basic Local Alignment Search Tool (BLAST) in the National Center for Biotechnology Information (NCBI) website was used to compare the 16S rRNA gene sequences with those in the NCBI database in order to know the closest relatives as described by Al-Jaradi et al. [21]. The evolutionary history was inferred using the neighbor-joining method, and the evolutionary distances were computed using the maximum composite likelihood method. Evolutionary analyses were conducted in MEGA11.

### 2.5. Evaluation of Putative Bacterial Biocontrol Activity against *A. alternata* under Greenhouse Conditions

The experiments were conducted in a randomized complete block design of three blocks with ten bacterial biocontrol treatments and respective positive and negative controls. Three GLP 2 Rosecoco bean seeds were planted in twelve pots, and each treatment was replicated three times. The bean seeds were watered every seven days. Two hundred milliliters of bacterial biocontrol suspensions was prepared by diluting a 24 h bacterial culture to a cell concentration of 1 × 10^8^ CFU/mL and sprayed to runoff on the upper and lower leaves of 21-day-old bean plants. Three hours later, an *A. alternata* spore suspension containing 1 × 10^6^ spores/mL was spotted on the second, third, and fourth leaves using sterile cotton buds. The positive control plants were sprayed with a commercial Bonide sulfur fungicide according to the manufacturer's instructions, while the negative control plants were sprayed with sterile distilled water following *A. alternata* application. We sprayed the plants with biocontrol suspensions on the 15th, 30th, and 45th day postinfection with *A. alternata*. Disease incidence was recorded as the percentage of diseased leaflets collected per treatment. Disease severity was scored and recorded using the leaf symptom index (LSI) at 15, 30, and 45 days beginning at fifteen days after inoculation using a 0–5 rating scale as follows: 0 = no symptoms in all leaves, 1 = only 1 leaf partially wilted, 2 = 2-3 wilted leaves, 3 = 2/3 wilted, 4 = all leaves wilted, and 5 = plant dead. The lesions were classified as follows: no lesions = 1, lesions >30% = 2, lesions >60% = 3, lesions >90% = 4, and plant death = 5 [22].

A total of 3 LSI estimations were made per treatment. Leaf disease incidence and leaf numbers were determined at the close of the field experiment. Analysis of variance was used to assess the effectiveness of bacterial biocontrol against *A. alternata*, and the mean differences between controls and diseased plants were compared using Duncan's multiple range test at *P*=0.05. To evaluate the colonization efficiency of the bacterial biocontrol and pathogen, we reisolated and characterized both using standard microbiological procedures as described above. The experiment was repeated twice under the greenhouse conditions of 27–30°C.

## 3. Results

### 3.1. Morphological Characterization of *A. alternata*

**Figure 1:**
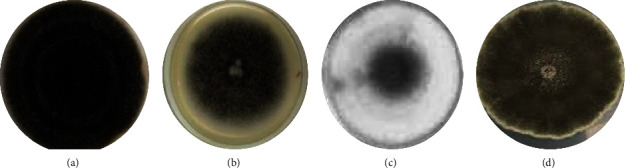
Morphological characteristics of select fungal isolates. (a) *A. alternata* isolate dark grey in color; (b) *A. alternata* isolate light grey in color; (c) *Alternaria* spp. isolate white in color; (d) *Alternaria* spp. isolate light brown in color.

Five unique fungal isolates were obtained from diseased leaf samples. All the fungal isolates grew within one week at 28°C and showed different morphologies (Figure 1).

### 3.2. Morphological Characterization of Bacterial Biocontrol Isolates

Of twenty-seven bacterial isolates recovered, eight showed antagonistic activity against *A. alternata*, and these were selected for further analysis. Morphological characterization was performed on the basis of color, elevation, form, and margin (Table 1).

### 3.3. Physiological and Biochemical Characterization of Bacterial Isolates

**Figure 2:**
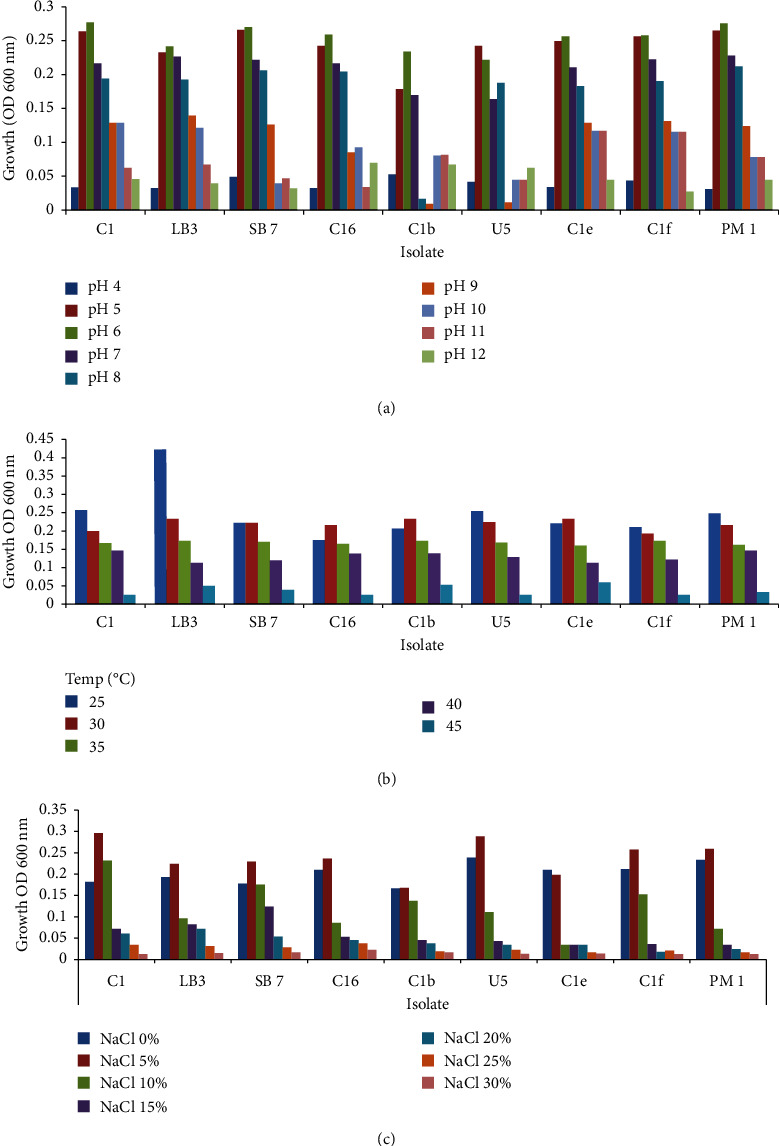
Growth of bacterial biocontrol isolates at different physiological conditions: (a) pH; (b) temperature; (c) sodium chloride.

These isolates could grow between pH 4 and 12 with the optimal pH recorded at pH 6 except isolate U5 (pH5). Growth decreased when pH was above 8 (Figure 2(a)). All isolates were able to grow within temperatures of 25–45°C. The optimum temperature for the isolates was 25°C with the least growth at 45°C (Figure 2(b)). The optimum salt concentration for the isolates was 5%, while growth decreased towards 30% (w/v) as shown (Figure 2(c).

The isolates grew on various substrates as shown (Table 2). All isolates utilized starch as a sole carbon source, while isolates C16 and C1c utilized all substrates.

Isolate C1e utilized starch and skimmed milk, while isolate LB3 utilized skimmed milk, tributyrin, citrate, and phosphate. Isolate C1 utilized starch, CMC, skimmed milk, tributyrin, and phosphate, while isolate C1b utilized starch, skimmed milk, tributyrin, and phosphate. Isolate C1f utilized starch and tributyrin only, while U5GH utilized starch only.

### 3.4. Molecular Characterization of Fungal and Bacterial Isolates

A total of 26 fungal isolates were isolated from diseased leaves of the GLP 2 Rosecoco bean variety. BLAST analysis showed that 5 of them were affiliated to the genus *Alternaria* with varying levels of relatedness (Table 3).

Eight bacterial isolates were identified as having antagonistic activity against the fungal isolates (Table 4).

**Figure 3:**
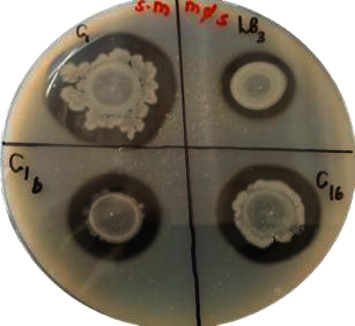
Antagonistic activity of selected bacterial isolates against *A. alternata.*

The isolates were closely clustered to the genus *Myroides, Bacillus, Paenibacillus,* and *Brevibacillus*, and they showed activity against *A. alternata* with some showing strong clear zones and other moderate or weak clear zones (Figure 3).

### 3.5. Evaluation of Antagonistic Bacterial Isolates against *A. alternata* under Greenhouse Conditions

**Figure 4:**
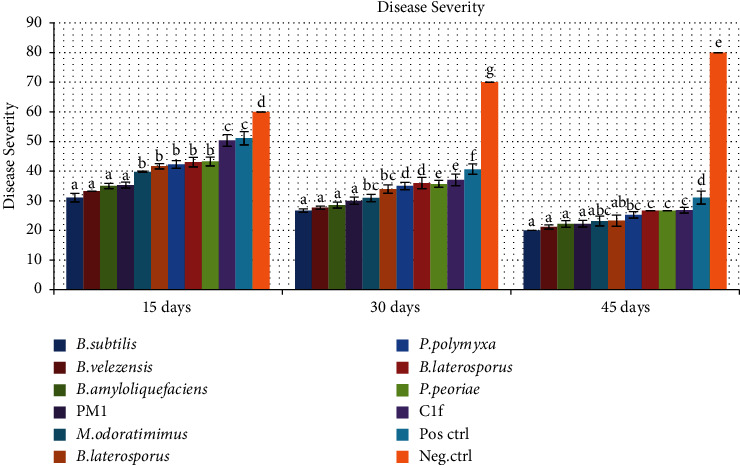
Disease severity of Alternaria leaf spot in GLP 2 Rosecoco bean variety plants at 15, 30, and 45 days postinfection. Values followed by the same values are not significantly different (*P* < 0.001).

Isolates LB3 (*Bacillus subtilis*), C1b (*B. velezensis*), C1e (*B. amyloliquefaciens*), C1 (*Myroides odoratimimus*), C16 (*B. laterosporus*), SB7a (*P. polymyxa*), and PM1 (*P. peoriae*) showed reduced leaf disease severity and incidence at *P* < 0.001. The highest disease severity was recorded at 15 days for Bonide sulfur-treated plants and untreated plants, respectively (Figure 4). The lowest disease severity was recorded at 45 days for plants treated with *Bacillus subtilis* and *Bacillus velezensis*, respectively (Figure 4).

**Figure 5:**
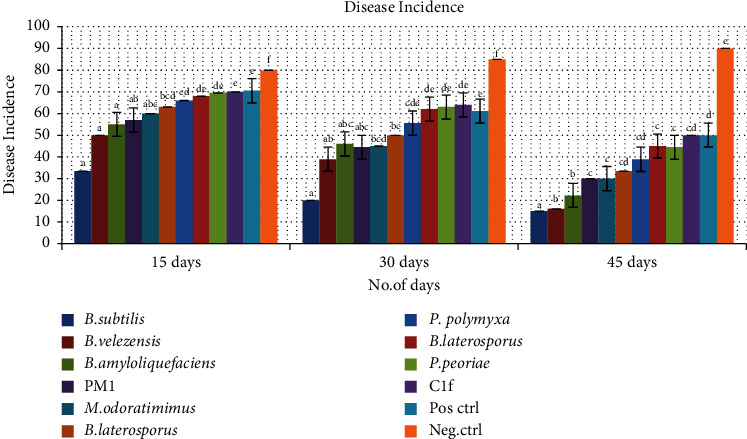
Disease incidence of Alternaria leaf spot in GLP 2 Rosecoco bean variety plants at 15, 30, and 45 days postinfection. Values followed by the same values are not significantly different (*P* < 0.001).

The highest disease incidence was recorded at 15 days for Bonide sulfur-treated plants and untreated plants (Figure 5). The lowest disease incidence was recorded at 45 days for plants treated with *B. subtilis* and *B. velezensis* (Figure 5). At 30 days, the highest disease severity was recorded for Bonide sulfur-treated plants and untreated plants, while the least disease severity was recorded for plants treated with *B. subtilis* and *B. velezensis*. There was a significant difference (*P* > 0.001) in disease incidence and severity among the bacterial biocontrol treatments and the untreated plants (Figures 4 and 5).

#### 3.5.1. The Effect of *B. subtilis*, Positive Control (Bonide Sulfur), and Negative Control (Untreated Plants) on GLP 2 Bean Leaf Appearance under Greenhouse Experiment

**Figure 6:**
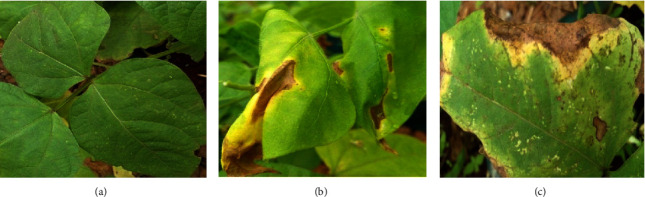
Pot experiment showing the effect of *B. subtilis*, positive (Bonide sulfur), and negative control (untreated) on GLP 2 bean leaf appearance. (a) *B. subtilis*-treated leaves were not spotted; (b) Bonide sulfur-treated leaves were moderately spotted; light brown spots with a yellow halo around them; (c) untreated leaves were heavily spotted with light brown centers surrounded by dark concentric rings and yellow halos.

*B. subtilis*-treated leaves were not spotted, Bonide sulfur-treated leaves were moderately spotted with light brown spots and yellowing, while untreated leaves were heavily spotted turning with light brown centers surrounded by dark concentric rings and yellow halos as shown in Figure 6.

### 3.6. Reisolation and Molecular Characterization of Fungal Pathogen and Bacterial Biocontrol Isolates

Bacterial and fungal colonies were reisolated from GLP 2 bean leaves which were surface-sterilized and cut into tiny pieces of about 2-3 mm. Leaf material was crushed and suspended in PBS, and serial dilution was conducted. One hundred microlitres of dilutions 10^−2^ and 10^−4^ was plated on PDA and TSA plates for fungal and bacterial growth, respectively. Three bacterial isolates and two fungal isolates were reisolated and identified as shown (Table 5).

## 4. Discussion

This study explored the potential of isolates recovered from the rhizosphere to control brown leaf spot disease in GLP 2 bean leaves. By using cultural and molecular techniques, we determined that *A. alternata* was the causative agent of the brown spots on diseased GLP 2 bean leaves. We hypothesized that rhizospheric bacteria present in bean fields could have potential biocontrol properties. We characterized seven unique biocontrol isolates identified as *B. subtilis*, *B. velezensis*, *B. amyloliquefaciens*, *M. odoratimimus*, *B. laterosporus*, *P. polymyxa*, and *P. peoriae* based on their ability to inhibit the growth of *A. alternata*. *B. subtilis* and *B. velezensis* showed strong inhibitory effects. These findings are in agreement with those of Govardhani et al. [23] who reported that *B. subtilis* reduced *A. alternata* radial growth, while Toral et al. [24] reported that *B. velezensis* suppressed *in vitro* growth of pathogenic *A. alternata* by about 40%. According to Desmyttere et al. [25], *Bacillus* spp. produce several lipopeptide metabolites that adhere to and disrupt target fungi's cell membranes. *In vitro*, *Bacillus spp.* produce hydrolytic enzymes like lipases and proteases that break down fungi's cell walls [24].

The isolates had the highest growth at pH 5.0–6.0 and decreased growth at pH values 7, 8, 9, and 10. Enzymatic activity increases up to the optimum pH and decreases beyond optimum pH. Drastic variation in pH can destroy the plasma membrane of bacteria and prevent enzyme activity and membrane transport proteins, thus decreasing growth. This finding is in line with Trimulyono [26] who reported that most *Bacillus* spp. isolated from the soil are neutrophils growing optimally at one or two pH below pH 7. At optimum temperatures, all biocontrol isolates grew best at 25 and 30°C and least at 45°C. Metabolic activity increases at optimum temperatures [26]. The bacterial enzymes become denatured at temperatures above 45°C, resulting in a decrease in growth. Based on this, it is likely that our biocontrol isolates are mesophiles, which grow best at a temperature of 25°C. We observed that the ability of our biocontrol's to withstand a range of 10 and 15% NaCl indicated that they are slightly halophilic.

Eight out of ten biocontrol agents with antagonistic effects on agar plates were similarly efficient in reducing leaf disease severity and incidence in GLP 2 bean plants. Our data showed that rhizospheric bacteria could serve as biocontrol agents for Alternaria leaf spot instead of commercial Bonide sulfur. According to Trimulyono [26], *Bacillus* spp. are a potential bioagent against Alternaria fruit rot. The bacterial bioagents inhibit pathogen development by producing antibiotics or enzymes, competing with pathogens, and colonizing rapidly. In addition, Lastochkina et al. [27] found that harvested tubers treated with *B. subtilis* were less likely to be infected by *Alternari*a and *Fusarium* spp. Wang et al. [28] reported *Bacillus* species produce cyclic lipopeptides (surfactins) that suppress bacterial and fungal pathogens. Finally, we report that rhizospheric *B. subtilis* and *B. velezensis* isolated from bean growing soil can be used as bioagents to control *A. alternata*. To the best of our knowledge, these results demonstrate for the first time that rhizospheric *B. subtilis* and *B. velezensis* can act as bioagents in the control of *A. alternata* in GLP 2 beans. Our study, however, did not combine different biocontrols to assess their efficacy. Future studies should combine biocontrol agents to test their efficacy against Alternaria leaf spot. The need for sustainable bean production will be met in part by adopting use of biocontrol strategies in combination with other control methods.

## 5. Conclusion

Of eight identified bacterial biocontrol isolates, rhizospheric *B. subtilis and B. velezensis* displayed the highest antagonistic activity against *A. alternata*. The two *Bacillus* species could be excellent candidates for offering long-term biocontrol potential for the management of Alternaria leaf spot in GLP 2 bean plants. Future studies should combine biocontrol agents to test their efficacy against Alternaria leaf spot.

## Figures and Tables

**Table 1:** Morphological characterization of bacterial biocontrol isolates.

Serial number	Color	Elevation	Form	Margin
C1e	Yellow	Flat	Round	Entire
LB3	White	Flat	Irregular	Undulated
SB7a	Yellow	Flat	Round	Undulated
PM1c	White	Flat	Round	Entire
C1	White	Flat	Irregular	Undulated
C1c	White	Flat	Round	Entire
C1b	White	Flat	Irregular	Undulated
C1f	White	Flat	Irregular	Undulated

**Table 2:** Substrate utilization by the various bacterial isolates.

Isolate	Starch	CMC	Skimmed milk	Tributyrin	Citrate utilization	Phosphate solubilization
C1e	+++	++	−	−	++	++
LB3	++++	−	++	++	+++	+++
SB7a	++	++	++	++++	−	−
PM1c	++	−	−	++	++	++
C1	++	++	++	++++	−	++
C1c	++	+++	++	++	+++	+++
C1b	++++	−	++	++	−	++
C1f	++	−	−	++	++	++
U5GH	++	−	−	−	−	−
C16	++	++	++	++	++	++

*Note*. (++) indicates low activity (growth took >3 days), (+++) denotes moderate activity (growth took 2-3 days), (++++) indicates high activity (growth took <1 day), and (−) sign indicates absence of visible enzyme activity.

**Table 3:** Phylogenetic analysis and identification of fungal isolates isolated from leaves of bean plants displaying leaf spot symptoms.

Isolates	Next neighbor in BLAST	Accession	% identity
U5	*Alternaria* sp. MG3 strain HC2	MT626575.1	100
C2	*Alternaria* sp. strain MG3 strain HC2	MT626575.1	100
PM2	*Alternaria* sp. strain HC2	MT644140.1	100
C16	*Alternaria alternata*_99	ON22688	99
LB3	*Alternaria alternata*_92	ON361375.1	92.16

**Table 4:** Antagonistic activity of the bacterial isolates against *A. alternata*.

Isolate	Closest relative	Accession	% identity	Activity against fungal pathogens				
				PM2	U5	LB3	C2	C16
C1	*Myroides odoratimimus* YRL08	EU373415.1	98.84	++	++	++	++	++
LB3	*Bacillus subtilis* strain NN29	MT114572.1	99.32	++++	+++	++++	+++	++++
SB7a	*Paenibacillus polymyxa* DSM36	NR114810.1	98.5	+	+	+	+	+
C16	*Brevibacillus laterosporus BL-2*	DQ371289.2	99.77	+	+	−	−	−
C1b	*Bacillus velezensis strain PgBE8*	MH211270.1	97.33	++++	+++	++++	+++	++++
U5GH	*Brevibacillus laterosporus* LHC95	KC951909.1	98	++	+	+	+	+
C1e	*Bacillus amyloliquefaciens* SWM1	JN851189.1	99.32	+++	+++	+++	+++	+++
PM 1c	*Paenibacillus peoriae* strain	MN826537.1	85.05	+	−	+	−	+

+, low activity; ++, medium activity; +++, high activity; ++++, extremely high activity; −, no activity.

**Table 5:** Molecular identification of reisolated fungal and bacterial isolates.

Isolate code	Closest strain	Accession	Identity (%)
C1	*Bacillus subtilis*	MT114572.1	98.84
C2	*Bacillus velezensis*	MH211270.1	97.33
C3	*Bacillus amyloliquefaciens*	JN851189.1	99.32
A1	*Alternaria alternata*_99	ON22688	99.62
A2	*Alternaria alternata*_92	ON361375.1	92.12

## Data Availability

Data will be provided from the corresponding author on reasonable request.
